# Does optimizing Choose to Move – a health-promoting program for older adults – enhance scalability, program implementation and effectiveness?

**DOI:** 10.1186/s12966-024-01649-9

**Published:** 2024-12-18

**Authors:** Lindsay Nettlefold, Heather M. Macdonald, Joanie Sims Gould, Adrian Bauman, Zoe Szewczyk, Heather A. McKay

**Affiliations:** 1 Active Aging Research Team, University of British Columbia, Vancouver, BC Canada; 2 Department of Family Practice, University of British Columbia, Vancouver, BC Canada; 3 Sydney School of Public Health, University of Sydney, Sydney, NSW Australia

**Keywords:** Optimization, Adaptation, Scale-up, Implementation, Health promotion, Older adults, Physical activity, Loneliness, Social isolation

## Abstract

**Background:**

Investment in scale-up and sustainment of effective health-promoting programs is often hampered by competing demands on scarce health dollars. Thus, optimizing programs to reduce resource use (e.g., delivery costs) while maintaining effectiveness is necessary to promote health at scale. Using a phased approach (2015–2024), we adapted and scaled-up an evidence-based, health-promoting program for older adults (Choose to Move; CTM). For CTM Phase 4 we undertook a systematic, data-driven adaptation process to reduce resource use. In this paper we: 1) describe the CTM Phase 4 program (‘CTM Phase 4’) and assess its 2) implementation and 3) effectiveness.

**Methods:**

For CTM Phase 4 (30-min one-on-one consultation and 8, 60-min group meetings with an activity coach), we reduced activity coach hours by 40% compared to Phase 3. To evaluate effectiveness of CTM Phase 4 we conducted a type 2 hybrid effectiveness-implementation study involving 137 programs (1126 older adults; 59–74 years, 75 + years) delivered by 29 activity coaches. We assessed implementation indicators (e.g., dose, fidelity, adaptation, participant responsiveness, self-efficacy) via survey in activity coaches and older adults. We assessed older adults’ physical activity (PA), mobility, social isolation, and loneliness before and after (0, 3 months) the program.

**Results:**

Implementation indicators demonstrated that CTM Phase 4 was delivered successfully. Post-intervention, PA (+ 1.5 days/week; 95% CI 1.3, 1.6), mobility limitations (-6.4%), and scores for mobility (+ 0.7; 95% CI: 0.4, 1.3), social isolation (+ 0.69; 95% CI: 0.50, 0.89), and loneliness (-0.24; 95% CI: -0.34, -0.13) were improved in those < 75 years. Among those ≥ 75 years, PA (+ 1.0 days/week; 95% CI, 0.7, 1.2), mobility score (+ 1.1; 95% CI: 0.4, 1.8), and social isolation score (+ 0.31; 95% CI: 0.002, 0.61) were improved post-intervention. Overall, participant-level benefits were comparable to those observed in Phase 3.

**Conclusions:**

CTM was co-designed as a flexible program, adapted over time based on user group needs and preferences. This flexibility enabled us to reduce activity coach delivery hours without compromising implementation or benefits to older adults’ health. Optimizing effective health-promoting programs to enhance their scalability and sustainability provides an important pathway to improved population health.

**Trial Registration:**

ClinicalTrials.gov, NCT05678985. Registered 10 January 2023 – Retrospectively registered, https://clinicaltrials.gov/study/NCT05678985.

**Supplementary Information:**

The online version contains supplementary material available at 10.1186/s12966-024-01649-9.

## Background

To improve health at the population level, researchers and health practitioners must better understand how to scale-up [1] and sustain [2] effective health-promoting interventions within meagre public health budgets. Scaling-up is the process of expanding delivery of evidence-based interventions (EBIs) to reach more of the eligible population (i.e., those for whom the intervention was designed) [3]. Sustaining is the process of continued delivery of the EBI so that benefits persist [4]. Implementation science has significantly advanced the study of initial adoption and implementation of EBIs across a range of community and health care settings [5]. However, less attention has been paid to how to sustain EBIs [2, 6], particularly at scale [7]. This is “one of the most significant translational research problems of our time” [6] and a “persistent challenge across a range of settings and service delivery sectors, and across health behaviours and outcomes” [2]. When EBIs are not sustained, benefits to participants and organizations are not maintained, investments of time and resources are wasted [2], and research/public health partners may lose the trust and support of communities [8].

To help sustain EBIs, scale-up process guides highlight the critical need to monitor scale-up as it progresses, with a targeted focus on improving the *efficiency* of program delivery [9]—that is, maintaining EBI effectiveness at a lower cost (or increasing effectiveness for the same cost) [10]. This aligns with the concept of *optimization*, defined for the public health context as “a deliberate, iterative and data-driven process to improve a health intervention and/or its implementation to meet stakeholder-defined public health impacts within resource constraints” [11].

Since 2015, we have worked with delivery partner organizations (DPOs) across British Columbia (BC) Canada, to expand (horizontal scale-up [3]) delivery of an effective, health-promoting model called Choose to Move (CTM). Using an integrated and iterative knowledge translation (iKT) approach [12], CTM progressed from a translational formative evaluation (2015) [13, 14], through pilot (Phase 1; 2016), initial scale-up (Phase 2; 2016–2017) [15–17], broad scale-up (Phase 3; 2018–2020) [18] and virtual delivery (due to COVID; 2020) [19, 20] phases. Prior to Phase 3, we systematically adapted CTM for ‘best fit’ and to support broad scale-up, based on feedback from older adult participants and delivery partners [21]. Prior to Phase 4 (the focus of this study) we conducted a systematic and data-driven adaptation process to optimize the CTM program (i.e., maintain program effectiveness while reducing resource use). For CTM, the bulk of direct program delivery costs stem from program delivery staff (activity coaches); we adapted CTM to reduce the number of activity coach hours while retaining fidelity to the program’s *core functions.* Core functions are the essential elements (i.e., specific behaviour change techniques such as goal setting and action planning) of the intervention that drive change and make the EBI ‘work’ [22]. Therefore, our study has three objectives:

1. to describe the CTM Phase 4 program (‘CTM Phase 4’), optimized to maintain effectiveness with reduced activity coach delivery hours;
2. to assess whether CTM Phase 4 is implemented with fidelity (primary implementation outcome) and describe implementation indicators associated with delivery of CTM Phase 4 (dose delivered/received, adaptation, participant responsiveness, self-efficacy; secondary implementation outcomes); and
3. to evaluate whether CTM Phase 4 improved older adults’ physical activity (primary effectiveness outcome), mobility, and feelings of social isolation and loneliness (secondary effectiveness outcomes).

## Methods

### Choose to Move

The CTM model is comprised of the CTM program (https://choosetomove.ca) and a suite of implementation strategies that support program delivery. The CTM program is choice-based, coach- and peer-supported, and designed for low active (< 150 min/week moderate-to-vigorous physical activity (PA)) community-dwelling older adults. The CTM program supports participants to choose physical activities that align with their personal preferences, health status, and available resources [15, 16, 18]. We describe previous phases of CTM in detail elsewhere [13–15, 18, 21].

### Optimizing the Choose to Move program

Our approach to optimize the CTM program for Phase 4 focused on maintaining fidelity to the ‘core functions’ (i.e., the specific behaviour change techniques [23]) of the program while reducing program delivery hours by activity coaches. The multi-step process (Table 1) was modeled after our adaptation for best ‘fit’ between Phases 2 and 3 [21]. Briefly, this approach was informed by the Planned Adaptation Model [24], the National Cancer Institute adaptation framework [25] and iKT principles [12]. We used program delivery data and activity coach job descriptions/contracts from Phase 3 to inform adaptations. In Phase 3, activity coaches were contracted by DPOs for 67 h/program. During COVID we rapidly adapted the CTM program with our primary DPOs for virtual delivery (CTM-Virtual, 2020) [19, 20]. Therefore, for the final CTM Phase 4 program we incorporated elements of virtual delivery. The UBC Research Ethics Board approved all study procedures related to the optimization process (H15-02522).

**Table 1:** Overview of the multistep adaptation process

Step	Who	Data Source	Sample Questions
**1) Identify opportunities to optimize CTM for cost**			
a) Review existing data	CTM project team	Delivery partner contracts	• Number of contract hours to deliver a single program
	Older adults	Phase 1–3 program feedback (program mid-point; closed-ended responses)	• How useful did you find 1) telephone check-ins with your activity coach, 2) CTM group meetings, and 3) program materials?; How satisfied were you with CTM overall?; [Phase 3 only] What was your favourite part of CTM?
	Activity coaches	Phase 1–3 program feedback (program mid-point and upon completion; open and closed-ended responses)	• [Mid-point] What challenges did you face during the first 3 months? • [End of program] What challenges did you experience delivering this CTM program? What worked well for you this time? Any new challenges they had not experienced before? Any major differences delivering CTM to this group of participants?
		Phase 3 semi-structured interviews	• Semi-structured interviews (*n* = 9; summer 2019) focused on implementation facilitators and barriers
b) Collect new data	Older adults	Semi-structured interview	• Semi-structured interviews (*n* = 10; Oct 2019) focused on favourite/least favourite part(s) of CTM and preference/relative importance of group meetings vs. telephone check ins
	Activity coaches	Focus groups	• Focus groups (*n* = 3; 8 participants total; Oct—Nov 2019) focused on favourite/least favourite part(s) of CTM; what they would cut/keep/change if only 50% of funds available; feedback on check-ins
**2) Develop a prototype**			
	CTM project team	Data from step 1	• Internal project team meetings (*n* = 3; 6–10 attendees; Nov—Dec 2019) to integrate data from step 1 and develop prototypes with fewer estimated delivery hours; discuss risks, opportunities, and mitigation strategies for prototypes; select leading candidate prototype
**3) Validate prototype**			
	Activity coaches	Focus group	• Focus group (*n* = 1; 3 participants Jan 2020) focused on overall thoughts of the proposed changes (structural and content)
**4) Rapid adaptation due to COVID-19**			
	Leads of DPOs	Meetings with field notes	• Meetings (*n* = 18; Mar-Apr 2020) focused on whether they could continue to deliver CTM given current COVID-19 context; challenges and potential solutions; an adapted CTM (at home) virtual model; what support was required
**5) Incorporate learnings from virtual delivery of CTM = final phase 4 program**			
	CTM project team	Data from step 4	• Internal project team decision to continue successful elements of CTM virtual model (e.g., e-newsletter; option to deliver virtually)

*CTM* Choose to Mov﻿e, *DPO* Delivery partner organizations

### Choose to Move Phase 4

The CTM Phase 4 program retained all core functions (Table S1) but required fewer activity coach hours to deliver; activity coaches were contracted by DPOs for 40 delivery hours/program (40% reduction). Within the CTM Phase 4 program participants engaged in 2 program components over 3 months (compared with 6 months for Phase 3): a 30-min one-on-one consultation with an activity coach (compared with 60-min for Phase 3) and 8 group meetings with other CTM participants (compared with 5 group meetings for Phase 3). Telephone check-ins were eliminated for Phase 4. CTM Phase 4 is delivered in person in community settings as in Phase 3 [13–15, 18, 21], or virtually via Zoom or GoToMeeting platforms as in CTM-Virtual [19, 20]. For CTM Phase 4 we also offered an e-newsletter to participants. We describe differences between CTM Phase 3 and 4 in Table 2 and provide a detailed description of CTM Phase 4 using the TIDieR [26] checklist (Table S2).

**Table 2:** Differences between CTM Phase 3 and 4 programs

	**CTM Phase 3**	**CTM Phase 4**
Activity coach delivery hours	• 67 h per program	• 40 h per program
Program length	• 6 months	• 3 months
Format	• Information session 1–2 weeks prior to program start • Initial 60-min consultation one week prior to group meeting 1 • 5 group meetings (in-person) • 6 check-ins (telephone, email, in-person)	• Information Session: Same as Phase 3 • Initial 30-min consultation between group meetings 1 & 2 • 8 group meetings (in-person; virtual; hybrid) • 0 check-ins
Intervention activity: *Group meetings*	• Health topics covered in group meetings 1. Physical activity & social connection 2. Healthy weight management & nutrition 3. Stress & anxiety 4. Brain health & preventing injury 5. Revisit your goals & celebrate! • Prescribed movement breaks during meetings • Group meeting slides prescriptive for group and paired discussions; contact information formally included in each CTM participant group • No group challenges • No peer check-ins • No ‘check-in’ newsletter	• Health topics covered in group meetings 1. Welcome and goal setting 2. Physical activity & social connection 3. Incidental physical activity 4. Goals revisited 5. Nutrition 6. Falls prevention 7. Stress management & brain health 8. Goals and celebration • Prescribed movement breaks during meetings (in-person); none prescribed during virtual delivery of meetings for safety reasons, though activity coaches invited to encourage participants to get up and move around at some point during meeting • Group meeting slides: Similar to Phase 3 • Group check-ins during meetings • Group challenges included at the end of each group meeting • Optional peer check-ins • Optional ‘check-in’ newsletter (bi-weekly)
Intervention activity: *Check-ins*	• Six telephone check-ins (15 min, on average; once per month by phone, email, or in-person)	• No telephone check-ins • Core functions (e.g., goal setting, action planning, etc.) of the check-ins shifted to the group meetings (specifically, meetings 1, 4, and 8)

### Choose to Move implementation

CTM program delivery is guided by implementation [27] and scale-up [28] frameworks. Community-based DPOs offer the CTM program in community settings (e.g., recreation centres, neighbourhood houses) across the province. With funding from government, the Active Aging Society (AAS; www.activeagingsociety.org) provides broad functional and financial support to DPOs. A Central Support Unit [29] liaises directly with DPOs and uses a suite of implementation strategies (“methods or techniques used to enhance the adoption, implementation, and sustainability of a clinical program or practice” [30]) to support CTM program delivery and scale-up [15]. The Central Support Unit serves as a link between DPOs, the AAS and researchers from the Active Aging Research Team (University of British Columbia, UBC). In this paper we refer to the Central Support Unit and research team collectively as the CTM project team [29].

### Evaluating implementation and effectiveness of CTM Phase 4

#### Study design

To evaluate the CTM Phase 4 program we used a type 2 hybrid effectiveness-implementation study design [31] with mixed methods, as in previous phases [15, 16, 18]. We collected data pre- and post-intervention for all 137 Phase 4 programs with start dates between September 2020 and October 2022. The UBC Research Ethics Board approved all study procedures (H20-00780) for the evaluation and we retroactively registered the trial (Clinical trials registration: NCT05678985). Study reporting aligns with the Standards for Reporting Implementation Studies (StaRI) statement [32].

#### Participants

DPOs and activity coaches used a variety of approaches to recruit older adults to participate in CTM (e.g., online and print media, word of mouth). Eligible participants were low active (self-reported < 150 min/week of PA), community-dwelling individuals aged > 60 years, English speaking, and with no contra-indications to PA participation (PA Readiness-Questionnaire + [33], Get Active questionnaire [34], or physician clearance). All participants were invited to participate in the evaluation; however, as CTM is a free, community-run program, participation in the evaluation was not mandatory. All activity coaches who delivered programs in Phase 4 were informed of the evaluation at the time of hiring, and were invited to participate. All older adults and activity coaches who participated in the evaluation provided informed consent.

#### Implementation evaluation

Implementation indicators (fidelity, dose delivered, adaptation, and activity coaches’ self efficacy) were drawn from a proposed minimum data set [35]. To this list we added dose received (to capture varying attendance levels across older adult participants), participant responsiveness ("the degree to which the program stimulates the interest or holds the attention of participants" [27]) and facilitators and barriers to delivery. As in our previous studies [15, 16, 18] activity coaches completed: 1) a survey after training (one per coach to assess self-efficacy); 2) program feedback surveys (one per program to assess fidelity, dose delivered, adaptation, barriers and facilitators to delivery); and 3) participant engagement surveys (one per participant to assess dose received and perceived participant responsiveness). Older adults completed a program feedback survey at the end of the program (3 months; dose received, participant responsiveness). All activity coaches completed surveys electronically via REDCap [36, 37] as described previously [18]. For older adults who were unable to complete surveys online, we offered to mail a paper survey (with return envelope), or a trained research assistant to help them complete the survey over the phone. Almost all (96%) older adults completed surveys online.

#### Effectiveness evaluation

We replicated our previous survey-based measures: socio-demographics [15, 18], PA [38–40], mobility [41], and loneliness [43] in older adult participants [15, 18]. For social isolation [42] we replicated two of the three questions used previously and due to COVID, we separated the final question about attendance at meetings/programs into attendance at a) online programs and b) in-person programs. However, to facilitate comparison with previous phases (i.e., maintain a score range of 0-15) we created a single summary response for online and in-person programs before summing to create a final social isolation score. For example, a participant who reported attending online programs 1/week and in person programs 1/week (each scored as 4 on the 6-point Likert scale (0-5)) would be assigned a combined program score of >1/week (scored as 5). We added the mobility assessment tool short form (MAT-sf; [44, 45]). The MAT-sf is a 10-item computer-based, self-administered assessment that uses short video clips of specific tasks to illustrate a range of mobility-related challenges (e.g., walking uphill outdoors on uneven terrain, stepping over hurdles). Each video clip has an associated question (number of minutes, number of times, yes/no). The MAT-sf is scored via the software (range: 30–80; higher scores indicate higher percieved mobility) and shows good validity and reliability [46, 47]. Older adults who completed surveys by mail or phone (4%) did not complete the MAT-sf.

### Data analysis

We performed all analyses using Stata (version 13.1; StataCorp, College Station, TX). We first examined whether participants who were lost to follow-up (i.e., withdrew from the program or did not complete the evaluation) differed from participants who completed the program. We used two-tailed chi-squared or Fisher’s exact test for categorical variables (sex, age category, ethnicity, education, chronic conditions, mobility limitations, and subset participation) and analysis of variance for continuous variables (body mass index (BMI) and impact variables). Next, we used two-sided t-tests (for continuous variables) and chi-squared tests (for categorical variables) to compare socio-demographic characteristics at baseline between age groups (<75 years, ≥ 75 years).

As in our previous studies [15, 16, 18], we describe implementation indicators using percentages, means and ranges where appropriate. We use two-tailed chi-squared or Fisher’s exact tests to assess between-group differences. To address program effectiveness, we fit linear mixed effects models for each continuous outcome variable [PA (primary outcome), mobility (MAT-sf), social isolation, loneliness (secondary outcomes)] with time (0, 3 months) as a categorical predictor. We first fit an empty means random intercept model and tested whether random slopes improved model fit using likelihood ratio tests. In model 1, we included sex and age category (< 75 years, ≥ 75 years) as fixed effects. Model 2 included additional covariates: DPO, program cycle, baseline mobility limitation (yes/no), number of chronic conditions (0, 1, ≥ 2), education, and BMI. In both models, we added fixed effects sequentially and tested interactions with time after the addition of each fixed effect. With the exception of an age × time interaction, interactions were retained in the model only if the likelihood ratio test was significant (*p* < 0.05). We assessed model fit graphically using residual plots; plots indicated acceptable model fit. We also used chi-squared tests to assess differences in the proportion of participants with self-reported mobility limitations over time (0–3 months; secondary outcome) within each age group. We used a per-protocol approach, as participants who withdrew from the program also withdrew from the evaluation.

## Results

### Participants

Activity coaches initiated (i.e., at least one group meeting delivered) 137 CTM Phase 4 programs with 1278 participants between September 2020 and October 2022. Programs were delivered by 29 activity coaches (1–29 program(s)/activity coach) across 9 cycles that aligned with typical community centre programming start dates (Fall, Winter, Spring, Summer). Of the 137 programs initiated, one program was cancelled after the third group meeting when the facility closed due to COVID, and the activity coach did not move the group to a virtual platform. Two programs were merged with other groups due to small group size (< 6 participants), and one program was split into two after the first group meeting due to large group size (> 15 participants). Therefore, 135 programs were completed. Due to COVID, most (66%) programs were delivered virtually with fewer delivered in-person (29%) or as a combination of in-person and virtual (5%). Table 3 summarizes program characteristics.

**Table 3:** CTM Phase 4 program summary (*n* = 135 delivered programs)

	**Total**	**In-person**	**Virtual**	**Combined (in person & virtual)**
**Total number of CTM programs delivered** (start dates between Sept 2020 and Oct 2022)	135	39	89	7
**Number of CTM programs initiated by cycle** (Fall’20 / Winter’21 / Spring’21 / Summer’21 / Fall’21 / Winter’22 / Spring’22 / Summer’22 / Fall’22)	14 / 19 / 17 / 1 / 22 / 20 / 19 / 2 / 21	2 / 0 / 2 / 0 / 10 / 7 / 8 / 0 / 10	8 / 19 / 15 / 1 / 11 / 13 / 11 / 2 / 9	4 / 0 / 0 / 0 / 1 / 0 / 0 / 0 / 2
**Delivery organization** (BCRPA / YMCA)	91 / 44	32 / 7	53 / 36	6 / 1
**Number of sites delivering CTM**	83 programs were run through 27 sites (range: 1–17 programs /site) 52 virtual programs were not attached to a specific site (available to participants from multiple locations across BC)	39 in-person programs were run through 23 sites (range: 1–4 programs /site)	37 virtual programs were run through 8 sites (range: 1–17 programs /site) 52 virtual programs were not attached to a specific site and included participants from multiple locations across BC	7 programs were run through 6 sites (range: 1–2 programs /site)
**Number of activity coaches**	29^a^	18	18	5
**Number of programs per coach**	Range: 1–28 Median (IQR): 7 (3, 15) Mean (SD):11.3 (9.7)	Range: 1–10 programs per coach Median (IQR): 3 (2, 10) Mean (SD): 4.1 (3.6)	Range: 1–15 programs per coach Median (IQR): 7 (3, 15) Mean (SD): 8.5 (5.3)	Range: 1–3 programs per coach Median (IQR): 1 (1, 3) Mean (SD): 1.9 (1.1)
**Average duration of programs** (weeks; 11–12 is as planned)	Range: 7–17.3 Median (IQR): 10 (9, 11) Mean (SD):10.1 (1.4)	Range 8–17.3 Median (IQR): 11 (9.9, 11) Mean (SD): 10.5 (1.5)	Range 7–13.4 Median (IQR): 10 (9, 11) Mean (SD): 10.0 (1.3)	Range: 7–11 Median (IQR): 10 (10, 11) Mean (SD): 10 (1.4)

*BCRPA* British Columbia Recreation and Parks Association, *YMCA* Young Men’s Christian Association

^a^One coach leads programs for both BCRPA and YMCA

**Fig. 1:**
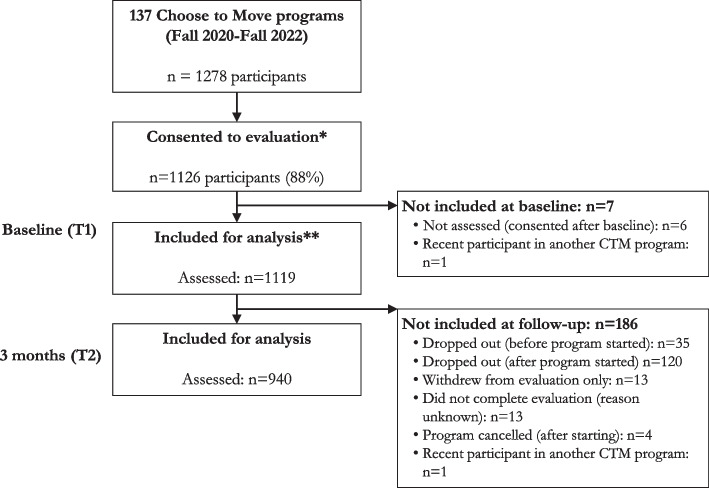
Participant flow through the study. For this study we included participants of Choose to Move programs delivered between Fall 2020 and Fall 2022 (program start dates between Sept 2020 – Oct 2022). *This includes 6 participants who consented late and did not provide baseline data **Participants who responded to at least one question on the evaluation survey are captured here as ‘included for analysis’. Exact numbers included for each variable are included in the text/tables

Of 1278 registered CTM participants, 1126 (88%) consented to be evaluated. We present the flow of participants through the study in Fig. 1 and baseline characteristics of the Phase 4 cohort in Table 4. Participants were mostly women (89%), self-identified as white (90%) and aged < 75 years (71%). Participants who withdrew from the study or were lost to follow-up at 3 months did not differ on any sociodemographic characteristics (Table S3). Where activity coaches were able to collect reasons for discontinuation, the most common reasons were not interested/did not see a benefit (31%), health concerns/injury (28%), scheduling difficulties (24%), family complications (8%), technology challenges (3%) and ‘other’ (6%).

**Table 4:** Baseline participant socio-demographic characteristics by age group in the Phase 4 Choose to Move cohort. Values are n (%) or mean (standard deviation). Sample size varies due to missing data

	**Total**	** < 75 years** **(*n* = 783)**^a^	** ≥ 75 years** **(*n* = 319)**^a^	***p*-value**
Participants, n (men / women / prefer not to answer)	1125 (128/993/4)	783 (76/707/0)	319 (50/269/0)	0.005
% (men)	11.4%	9.7%	15.7%	
Age, mean (SD) [*n* = 1102; range: 59–92]	71.5 (6.0)	68.4 (3.6)	79.1 (3.5)	
Age category, n (%) [*n* = 1102]				
< 75 years	783 (71.1%)^a^			
≥ 75 years	319 (29.0%)^a^			
Delivery partner, n (BCRPA / YMCA)	774 / 351	518 / 265	239 / 80	0.004
BMI, kg/m^2^				
Men (*n* = 125)	29.6 (6.0)	30.4 (5.7)	28.6 (6.5)	0.113
Women (*n* = 864)	30.3 (7.0)	31.0 (7.2)	28.4 (6.0)	< 0.001
Ethnicity, n (%) [*n* = 1106]				
White	991 (89.6)	687 (89.1)	292 (92.4)	
Asian	71 (6.4)	46 (6.0)	19 (6.0)	
Other	44 (4.0)	38 (4.9)	5 (1.6)	0.037
Educational attainment, n (%) [*n* = 1105]				
Secondary or less	196 (17.7)	121 (15.7)	71 (22.7)	
Some trade, technical school or college	339 (30.7)	241 (31.2)	93 (29.7)	
Some university	570 (51.6)	410 (53.1)	149 (47.6)	0.022
Chronic Conditions, n (%) [*n* = 1113]				
0	491 (44.1)	352 (45.4)	128 (40.5)	
1	322 (28.9)	225 (29.0)	93 (29.4)	
≥ 2	300 (27.0)	198 (25.6)	95 (30.1)	0.229
Mobility limitations (walk and/or stair), n (%) [*n* = 1117]				
Yes	454 (40.6)	489 (62.8)	157 (49.7)	
No	663 (59.4)	290 (37.2)	159 (50.3)	< 0.001
Self-rated health, n (%) [*n* = 1113]				
Very poor, poor or fair for age	642 (57.7)	485 (62.5)	147 (46.4)	
Good or excellent for age	471 (42.3)	291 (37.5)	170 (53.6)	< 0.001

*BCRPA* British Columbia Parks and Recreation Association, *YMCA* Young Men’s Christian Association

^a^23 participants did not provide their age so the sample size across these two columns does not match the total

### Program implementation

*Self-efficacy:* Of 29 activity coaches, 17 (59%) who delivered the CTM program for the first time (or the first time in an extended period) responded to the training feedback survey. Overall, activity coaches found the training useful (16/17 rated it 5 out of 5), felt confident they could apply it to their upcoming program (14/17 rated it 4 or 5 out of 5), and felt that they had the required resources/tools to deliver the program (15/17 rated it 4 or 5 out of 5).

*Dose delivered:* Across 135 programs, activity coaches delivered all (8/8) group meetings in 134 programs. In the remaining program the activity coach delivered six group meetings; they mistakenly followed the schedule for CTM-Virtual (6 group meetings).

*Fidelity to core functions:* Here we summarize the percentage of CTM programs where activity coaches reported fidelity to core functions at most (6–7) or all (8) group meetings. Activity coaches provided opportunities for group or peer check-ins (e.g., core functions of social support, shaping knowledge; Table S1) in 96% of programs, educational content in 100% of programs and opportunities to share resources in 98% of programs. Activity coaches encouraged peer check-ins (outside group meetings) in 85% of programs and shared a group challenge in 98% of programs. Reasons for non-fidelity to core functions varied. Generally, non-fidelity reflected activity coaches’ responsiveness to group needs/preferences and time constraints. For example, to respect existing social networks many activity coaches encouraged participants to check in with family and friends who were not participating in the program instead of peer check-ins. Concerns were also related to COVID and group dynamics. From the participant perspective, there were opportunities during some or all group meetings to interact (94%), learn about a health topic (96%) and share resources (94%). Across all programs, 23% of participants did peer check-ins outside of group meetings. Of these participants, 43% reported checking in with peers more than twice/month while others checked in twice (16%), once (14%) or less than once (27%) per month.

*Adaptation:* Adaptations during program delivery reported by activity coaches reflected responsiveness to the unique needs and dynamics of each group. For example, activity coaches added exercise or movement breaks to some virtual group meetings and eliminated them from some in-person group meetings due to time constraints. Activity coaches tailored other group meeting activities (e.g., removed icebreakers once groups were well connected, reduced focus on group and peer check-ins), and adjusted group meeting formats (e.g., using breakout rooms, meeting in the park instead of indoors) as they saw fit. Outside of group meetings, activity coaches spent time sourcing and sharing additional resources tailored to the group, sending additional email reminders, and reaching out to individuals by email or telephone if these participants missed meetings.

*Dose received:* Of the 1126 participants who consented to be evaluated, 1072 (95%) attended one or more group meetings. 735 (65%) attended 6 or more (≥ 75%) group meetings.

*Participant responsiveness:* Activity coaches considered 77% of participants very or extremely interactive; 97% of participants were deemed enthusiastic, interested, and engaged at most or all group meetings. Satisfaction with CTM was high among older adult participants; 85% reported feeling satisfied or very satisfied with the program. There were no differences in participant responsiveness by DPO or delivery mode (in person vs virtual).

### Program effectiveness

We present results of our impact evaluation in Table 5. Results were similar for minimally- and fully-adjusted models; below we focus on the fully-adjusted models for younger (< 75 years) and older (≥ 75 years) participants.

**Table 5:** Adjusted means (95% Confidence interval) for impact outcome measures by time point and age group

	**Months**	**Full sample** (***n* = 1126)**	** < 75 years** (***n* = 783)**^**a**^	** ≥ 75 years** (***n* = 319)**^**a**^	**p-value** **Full sample** **0–3 mos**	**p-value** ** < 75 yrs** **0–3 mos**	**p-value** ** ≥ 75 yrs** **0–3 mos**
**Physical activity** (# days/week > 30 min)	0	2.1 (2.0, 2.2)	2.0 (1.8, 2.1)	2.3 (2.1, 2.6)			
	3	3.4 (3.3, 3.5)	3.4 (3.3, 3.6)	3.3 (3.1, 3.6)	< 0.001	< 0.001	< 0.001
**Mobility** (n (%) reporting any limitation)	0	454 (40.6%)	290 (37.2%)	159 (50.3%)			
	3	324 (34.6%)	199 (30.8%)	121 (44.8%)	0.005	0.010	0.184
**Mobility** (MAT-sf score, 30–80)	0	51.4 (50.8, 52.0)	52.7 (52.0, 53.4)	47.9 (46.8, 49.1)			
	3	52.3 (51.7, 52.9)	53.4 (52.9, 54.3)	49.0 (47.9, 50.2)	< 0.001	< 0.001	0.002
**Social Isolation** (score, 0–15)	0	11.1 (10.9, 11.3)	10.8 (10.6, 11.0)	11.8 (11.4, 12.1)			
	3	11.7 (11.5, 11.9)	11.5 (11.3, 11.7)	12.1 (11.7, 12.4)	< 0.001	< 0.001	0.048
**Loneliness** (score, 3–9)	0	5.21 (5.09, 5.31)	5.35 (5.22, 5.48)	4.83 (4.62, 5.04)			
	3	5.04 (4.92, 5.15)	5.12 (4.98, 5.25)	4.86 (4.64, 5.07)	< 0.001	< 0.001	0.775

MAT-sf (mobility assessment tool-short form): higher score indicates greater perceived mobility; Social isolation: higher score indicates a larger social network; Loneliness: lower score indicates lower feelings of loneliness. ^a^23 participants did not provide their age so the sample size across these two columns does not match the total

*Physical activity:* Among younger and older participants, PA increased from baseline to 3 months (<75: + 1.5 days/week; 95% CI: 1.3, 1.6; ≥ 75: + 1.0; 95% CI: 0.7, 1.2).

*Mobility:* Among younger participants, prevalence of mobility limitations decreased by 6.4% from baseline to 3 months. Improvements in mobility among younger participants were also evident in the increased MAT-sf score at 3 months (+ 0.8; 95% CI: 0.4, 1.3). Among older participants, prevalence of mobility limitations did not change significantly from baseline to 3 months. However, MAT-sf score increased between baseline and 3 months among older participants (+ 1.1; 95% CI: 0.4, 1.8).

*Social isolation:* Among younger participants, social isolation score increased from baseline to 3 months (+ 0.69; 95% CI: 0.50, 0.89) indicating decreased feelings of social isolation. Older participants also demonstrated an increase in social isolation score (decreased isolation) between baseline and 3 months (+ 0.31; 95% CI: 0.002, 0.61).

*Loneliness:* Among younger participants, loneliness score decreased from baseline to 3 months (-0.24; 95% CI: -0.34, -0.13) indicating reduced feelings of loneliness. Among older participants, loneliness score did not change significantly between baseline and 3 months.

## Discussion

Effective, scalable, and sustainable solutions are urgently needed to address the dual epidemics of physical inactivity [48, 49] and loneliness [50, 51] that threaten older adults’ health and drive demand for health care services. However, government health departments and decision makers must navigate competing priorities and a relative scarcity of public health resources. In this study, we respond to this challenge by striving to reduce resource use, and thus cost, within the CTM program. We provide evidence that the adapted CTM Phase 4 program still ‘works’ compared with previous CTM phases as it enhanced older adult PA and mobility, while reducing their social isolation and loneliness. We discuss our new findings in the context of our previous work, and the field more broadly.

### CTM Phase 4 was implemented successfully

Despite the challenges presented by COVID, we collaborated closely with our community-based DPOs to adapt and deliver CTM Phase 4 virtually for two years during the pandemic (2020–2022). Only one program was cancelled due to COVID, when public health restrictions closed the facility. We consider this an implementation ‘*success’* that may reflect flexibility of the new Phase 4 delivery format—supporting both in-person and virtual delivery. As in CTM Phase 3 [18], activity coaches remained committed to the program—dose delivered (group meetings) in both Phases 3 and 4 was 100%. In previous Phases [42] we learned that the group meeting environment, and the relationship between activity coaches and participants, were key drivers of social connectedness outcomes. During COVID, these elements may have played an even greater role in supporting the social health of older adults who were isolating at home [19] further contributing to implementation success.

In CTM Phase 4, we reduced activity coach-participant contact hours while retaining all core functions (e.g., goal setting, social support). This adaptation did not appear to alter implementation by activity coaches or benefits to participants. Fidelity during CTM Phase 4 was similar to Phase 3 where fidelity to core functions ranged from 93–100%. Positive participant-level results in health promotion programs can be obtained with implementation above 60%; > 80% implementation is rare [27]. Thus, fidelity to core functions in CTM Phase 4 likely yielded benefits to participants. Given the key role of group meetings on social connectedness [42], the additional group meetings in CTM Phase 4 vs Phase 3 may have contributed to the positive impact of CTM Phase 4 on participants’ social health.

Scaling-up and sustaining EBIs are dynamic processes [52] that occur over the ‘life cycle’ of a program. As scale-up proceeds in new and varied contexts (e.g., populations, places), the program and implementation strategies need to be adapted to establish best ‘fit’. Across Phases 1, 2 and 3 [2016–2020], we scaled-up CTM across more diverse geographic areas of BC, (e.g., different urban centers, small northern towns, rural communities). As per our commitment to iKT [12], we conducted systematic adaptation processes that considered the context for delivery (e.g., demographics, geography) in these regions [21]. Program adaptation can help to achieve better reach, retention, feasibility and, as in the current study, to reduce the cost of resources associated with program delivery [53]. Although one school of thought is that adaptation is essential and inevitable [52], a ‘tension’ persists between adaptation and fidelity [54]. That is, the delivery system’s need to adapt an intervention to their specific context (e.g., geography, differing priorities, DPO capacity) while researchers often remain focused on delivering the intervention as planned [53]. However, we and others [22] suggest that it is important to consider *what* needs to be standardized within the program. For example, while a program’s *core functions* (i.e., behavior change techniques) should be maintained, the ‘*form*’ (i.e., how these techniques were delivered) can be altered to fit the implementation context. In theory, if a program’s core functions are retained, program effectiveness should not be compromised [22].

Therefore, for CTM Phase 4 we prioritized fidelity to core functions of the program, while allowing the ‘form’ to change. For example, we embedded behaviour change techniques associated with telephone check-ins in Phase 3 (i.e., revisiting goals, problem solving, social support) into group meetings in CTM Phase 4 [22] which allowed for fewer activity coach delivery hours. We know of only one other scaled-up health promotion trial [55] that formally evaluated outcomes after being optimized to reduce in-person contact. The optimization did not appear to negatively impact program outcomes, indicating that the elements (e.g., core functions) driving success were not altered [55]. That study, and our current work, highlight the importance of identifying interventions’ core functions. Ideally this should be done in the program design phase, however it is possible to do so retrospectively [56]. For a study to be effectively replicated, these details should be included in reporting using tools such as TIDieR [26].

We attribute successful implementation of CTM Phase 4 to four key factors. First, early on we actively engaged those who would be directly impacted by adaptations to CTM (i.e., older adults, activity coaches, DPO staff) in our decision making. This approach is a hallmark of our experience adapting and implementing CTM [21] over time and aligns with iKT principles [12]. Second, the flexible delivery format and technological gateway [20] allowed programs to start or migrate to virtual spaces to accommodate changing public health restrictions. Virtual options accommodated participant preferences and comfort and enabled participants with limited mobility, or who lived in more remote areas to participate. This, in turn, increased program reach. However, technology may be a barrier (e.g., access, skills) to virtual participation for some older adults [57, 58]. Third, the Central Support Unit provided tailored training, tools, and support before and during program delivery. These are all key elements of implementation and scale-up success [59]. The Central Support Unit also formed trusted relationships with DPOs; trusted relationships are considered essential as they contribute to enhanced motivation, capability and opportunity [60]. Fourth, despite significant challenges that COVID presented to not-for profit, health and recreation sectors, the commitment of our DPOs to older adults never wavered. They consistently prioritized delivery of CTM to older adults who were isolated during COVID. DPOs and activity coaches embraced training and adapted delivery of CTM, as new approaches (e.g., virtual delivery) were introduced. In so doing, they maintained safe and comfortable environments for older people to participate.

### CTM Phase 4 was effective

For CTM Phase 4 programs, we reduced the amount of one-to-one contact between activity coaches and participants and increased the number of group meetings. Despite this, participant-level increases in PA and mobility were similar to what we observed during the first 3 months of Phase 3 [18]. Overall, this suggests that the behaviour change techniques [23] were effective in our population (as per a recent review [61]) and that interactions between activity coaches and participants were sufficient to change behaviours. This, in turn, positively impacted participant-level outcomes across the 3-month program. The average increase in PA (+ 1.3 days/week over 30 min), although small, was meaningful for two reasons. First, participants engaged in very low levels of PA at baseline. Even small increases in PA have health benefits; these benefits are greatest in those who go from no or very low levels of PA to engaging in some PA [62–64]. Second, there is a vast (and expanding) literature on the health benefits of even small amounts of PA [62–64]. A recent systematic review noted meaningful health benefits can be gained from 75 min/week or less of moderate activity [63]; on average, participants’ PA levels at follow up exceeded this (3 days/week over 30 min). There are also established beneficial relationships between mobility, loneliness and social isolation and overall health [65–67]. Thus, the positive changes we reported for CTM participants begins to counter the widespread concern of loneliness and social isolation among older adults [51].

Between Phases 1–2 and 3 we observed a median ‘voltage drop’ or ‘scale-up penalty’ of 52.6% across outcomes [18]; however, we did not observe a further drop between Phases 3 and 4. This finding may be a function of slightly lower baseline PA in our Phase 4 sample (2.1 days/week; 95% CI: 2.0, 2.2) as compared with Phase 3 participants (2.5 days/week; 95% CI: 2.3, 2.6) allowing more room for change/improvements. The reason for lower baseline PA in our Phase 4 sample is unclear but may be related to facility closures during COVID restrictions. Reduced mobility limitations following program participation, were similar for Phase 3 (5.8%) compared with Phase 4 (6.0%), respectively. We attribute this to maintaining core functions, despite changes in program form, while recognizing the efforts of well-trained activity coaches and a highly skilled and experienced Central Support Unit (retained staff for 9 years). Importantly, participant-level outcomes did not differ between older adults who participated in-person compared with those who participated virtually. Thus, organizations and older adults might choose the delivery mode that best suits their needs and preferences.

### Strengths & limitations

We wish to highlight two major strengths of our study. First, CTM is one of only 6 interventions for older adults that was successfully scaled-up [68]. To our knowledge, CTM is the only health-promoting intervention that has had sustained government support for 9 years (since 2015). More than 5500 older adults have engaged in CTM since its inception [69]. Its longevity reflects a trusted two-way exchange of knowledge with community and funding partners, which was part of the impetus for adaptations we made and evaluated in the curent study. Second, we adopted an iKT process and engaged partners at every level to adapt, implement, and evaluate CTM. We committed to assess both implementation and effectiveness of adapted CTM programs, and to clearly communicate and discuss outcomes with partners. Again, this two-way flow of knowledge allowed us to retain the relevance of CTM for delivery partners and for older adults themselves, during a challenging health [COVID] crisis.

We also acknowledge that our study had several limitations. First, we focused on the role of activity coaches to deliver CTM programs. We acknowledge the fundamental role of DPOs and the Central Support Unit (and implementation strategies) in program delivery. As a next step, we aim to more fully consider CTM implementation strategies (i.e., assess the need for them, the form they take, their implementation, and their effectiveness). Second, we also focussed only on the cost of activity coaches to deliver CTM programs. We plan to conduct a formal economic evaluation in future that considers all costs (i.e., Central Support Unit, DPOs, training) of implementing CTM. Third, older adult participants were predominantly women who self-identified as white and lived in urban/densely populated areas. This highlights an inequity common across many older adults health promotion studies [70]. Thus, we acknowledge the need to adapt CTM so that it is more accessible and appropriate for a broader range of older adults (e.g., older men, older adults from more diverse ethnocultural groups, and those living in rural and remote communities). Fourth, we conducted a 3-month pre-post, pragmatic trial that lacks the robustness of randomized controlled trials. While our measures show evidence of validity and reliability [38, 39, 43, 46, 47], there is less evidence on responsiveness to change [40, 71], noted by others as well [72]. However, we feel this may be balanced by our study’s relevance in the ‘real world’. Fifth, COVID changed the underlying context and the environment for adapting and delivering CTM Phase 4. Older adults, activity coaches, and DPOs were all operating under unique, often stressful conditions that may have impacted baseline measures and/or their response to the program. However, results were promising in that delivery mode (virtual or in-person) did not influence program implementation or effectiveness.

## Conclusions

The research community must identify ways to adapt, optimize, scale-up, and sustain effective health-promoting interventions if we are to achieve health impact at the population level. If not, we undermine the substantial investment by government health partners and national granting agencies. Our body of work [13–21] illustrates that there is value in adopting an iKT approach [12] that comprises ongoing engagement, evaluation, and adaptation in close collaboration with end-users. We urge researchers and practitioners to monitor implementation, resource use, and effectiveness throughout the program lifecycle. These data can be used to optimize program delivery and resource use to manage cost as contexts inevitably change over time, and advocate for further investment as needed. As CTM enters its 10th year of delivery, we also note the need for this program (and all health-promoting programs) to reach more equity-deserving groups who may otherwise not have access to effective health-promoting initiatives. Finally, we reiterate the call to ‘begin with the end in mind’ [73, 74]. That is, to prioritize design, delivery and support of EBIs that are scalable and sustainable over the longer term.

## Supplementary Information

Supplementary Material 1: Table S1; Table S2; Table S3.

## Data Availability

The datasets analyzed during the current study are not publicly available as consent was not obtained for this. However, data are available from the corresponding author on reasonable request.

## Abbreviations

CTM: Choose to Move
DPO: Delivery partner organization
EBI: Evidence-based intervention
iKT: Integrated knowledge translation
