# Correction to: Does optimizing choose to move – a health‑promoting program for older adults – enhance scalability, program implementation and effectiveness?

**DOI:** 10.1186/s12966-025-01867-9

**Published:** 2025-12-20

**Authors:** Lindsay Nettlefold, Heather M. Macdonald, Joanie Sims Gould, Adrian Bauman, Zoe Szewczyk, Heather A. McKay

**Affiliations:** 1 Active Aging Research Team, University of British Columbia, Vancouver, BC Canada; 2 Department of Family Practice, University of British Columbia, Vancouver, BC Canada; 3 Sydney School of Public Health, University of Sydney, Sydney, NSW Australia

**Correction: Int J Behav Nutr Phys Act 21**,** 140 (2024)**

**https://doi.org/10.1186/s12966-024-01649-9**

After publication of the original article [1], we identified an error that impacted the analysis of one secondary outcome (social isolation). This error did not affect the primary outcome, nor any other secondary outcomes, and the overall conclusions of the study are not impacted.

In previous phases of Choose to Move (CTM) [2, 3], we assessed social isolation using 3 items, each rated on a 6-point Likert scale (0 to 5, where 0 is never and 5 is more than once a week) and created a score by summing the 3 items (range 0–15). With the onset of COVID, for CTM Phase 4 we modified the third item, which asked participants to report frequency of participation in meetings and programs. To distinguish between participation in-person and online meetings/programs, we asked participants to respond to two questions: how often they attended **online** meetings/programs and how often they attended **in-person** meetings/programs. We then created a summary score of the 4 items (range 0–20). However, for comparison with previous CTM phases (e.g., to estimate voltage drop with scale-up), we believe it is more appropriate to create a single combined response from the two items asking about participation in meetings and programs and use the combined response to calculate the social isolation score.

We re-analyzed the secondary outcome, social isolation, using the score calculated with the combined online/in-person response (range 0–15) and updated Table 5 accordingly. On average, social isolation scores were lower (due to the narrower range) at baseline and follow up than in the original analysis (range 0–20). The impact of CTM on social isolation remained statistically significant; however, the estimated magnitude of change was reduced, impacting the voltage drop calculation between Phase 3 and Phase 4. When we compare the magnitude of change in social isolation for Phase 4 (0–3 months) to the magnitude of change at intervention midpoint in Phase 3 (0–3 months), we now note a small voltage drop.

**Incorrect Table 5.**


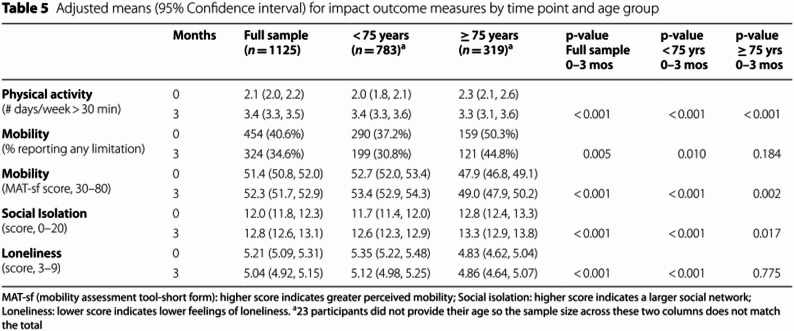


**Correct Table 5.**

**Table 5:** Adjusted means (95% confidence interval) for impact outcome measures by time point and age group

	Months	Full sample (*n* = 1126)	< 75 years (*n* = 783)	≥ 75 years (*n* = 319)	*p*-value Full sample 0–3 mos	*p*-value < 75 yrs 0–3 mos	*p*-value ≥ 75 yrs 0–3 mos
**Physical activity** (# days/week > 30 min)	0	2.1 (2.0, 2.2)	2.0 (1.8, 2.1)	2.3 (2.1, 2.6)			
	3	3.4 (3.3, 3.5)	3.4 (3.3, 3.6)	3.3 (3.1, 3.6)	< 0.001	< 0.001	< 0.001
**Mobility** (n (%) reporting any limitation)	0	454 (40.6%)	290 (37.2%)	159 (50.3%)			
	3	324 (34.6%)	199 (30.8%)	121 (44.8%)	0.005	0.010	0.184
**Mobility** (MAT-sf score, 30–80)	0	51.4 (50.8, 52.0)	52.7 (52.0, 53.4)	47.9 (46.8, 49.1)			
	3	52.3 (51.7, 52.9)	53.4 (52.9, 54.3)	49.0 (47.9, 50.2)	< 0.001	< 0.001	0.002
**Social Isolation** (score, 0–15)	0	11.1 (10.9, 11.3)	10.8 (10.6, 11.0)	11.8 (11.4, 12.1)			
	3	11.7 (11.5, 11.9)	11.5 (11.3, 11.7)	12.1 (11.7, 12.4)	< 0.001	< 0.001	0.048
**Loneliness** (score, 3–9)	0	5.21 (5.09, 5.31)	5.35 (5.22, 5.48)	4.83 (4.62, 5.04)			
	3	5.04 (4.92, 5.15)	5.12 (4.98, 5.25)	4.86 (4.64, 5.07)	< 0.001	< 0.001	0.775

Some text has also been corrected

**Incorrect Abstract**

Post-intervention, PA (+ 1.4 days/week; 95% CI 1.3, 1.6), mobility limitations (-6.4%), and scores for mobility (+ 0.7; 95% CI: 0.4, 1.3), social isolation (+ 0.9; 95% CI: 0.67, 1.17), and loneliness (-0.23; 95% CI: -0.34, -0.13) were improved in those < 75 years. Among those ≥ 75 years, PA (+ 1.0 days/week; 95% CI, 0.7, 1.2), mobility score (+ 1.1; 95% CI: 0.4, 1.8), and social isolation score (+ 0.5; 95% CI: 0.08, 0.86) were improved post-intervention. Participant-level benefits were comparable to, or greater (PA and social isolation in those < 75) than, those observed in Phase 3.

**Correct Abstract**.

Post-intervention, PA (+ 1.5 days/week; 95% CI 1.3, 1.6), mobility limitations (-6.4%), and scores for mobility (+ 0.7; 95% CI: 0.4, 1.3), social isolation (+ 0.69; 95% CI: 0.50, 0.89), and loneliness (-0.24; 95% CI: -0.34, -0.13) were improved in those < 75 years. Among those ≥ 75 years, PA (+ 1.0 days/week; 95% CI, 0.7, 1.2), mobility score (+ 1.1; 95% CI: 0.4, 1.8), and social isolation score (+ 0.31; 95% CI: 0.002, 0.61) were improved post-intervention. Overall, participant-level benefits were comparable to those observed in Phase 3.

**Incorrect Methods**.

We replicated our previous survey-based measures: socio-demographics [15, 18], PA [38–40], mobility [41], social isolation [42], and loneliness [43] in older adult participants [15, 18].

**Correct Methods**.

We replicated our previous survey-based measures: socio-demographics [15, 18], PA [38–40], mobility [41], and loneliness [43] in older adult participants [15, 18]. For social isolation [42] we replicated two of the three questions used previously and due to COVID, we separated the final question about attendance at meetings/programs into attendance at (a) online programs and (b) in-person programs. However, to facilitate comparison with previous phases (i.e., maintain a score range of 0–15) we created a single summary response for online and in-person programs before summing to create a final social isolation score. For example, a participant who reported attending online programs 1/week and in person programs 1/week (each scored as 4 on the 6-point Likert scale (0–5)) would be assigned a combined program score of > 1/week (scored as 5).

**Incorrect Results**.

Social isolation: Among younger participants, social isolation score increased from baseline to 3 months (+ 0.92; 95% CI: 0.67, 1.17) indicating decreased feelings of social isolation. Older participants also demonstrated an increase in social isolation score (decreased isolation) between baseline and 3 months (+ 0.47; 95% CI: 0.08, 0.86).

**Correct Results**.

Social isolation: Among younger participants, social isolation score increased from baseline to 3 months (+ 0.69; 95% CI: 0.50, 0.89) indicating decreased feelings of social isolation. Older participants also demonstrated an increase in social isolation score (decreased isolation) between baseline and 3 months (+ 0.31; 95% CI: 0.002, 0.61).

**Incorrect Discussion #1**.

Despite this, participant-level increases in PA and mobility, and reductions in social isolation and loneliness were similar to what we observed during the first 3 months of Phase 3 [18].

**Correct Discussion #1**.

Despite this, participant-level increases in PA and mobility were similar to what we observed during the first 3 months of Phase 3 [18].

**Incorrect Discussion #2**.

Between Phases 1–2 and 3 we observed a median ‘voltage drop’ or ‘scale-up penalty’ of 52.6% across outcomes [18]; however, we did not observe a further drop between Phases 3 and 4. This finding may be a function of slightly lower baseline PA in our Phase 4 sample (2.0 days/week; 95% CI: 1.8, 2.1) as compared with Phase 3 participants (2.3 days/week; 95% CI: 2.1, 2.6) allowing more room for change/improvements. The reason for lower baseline PA in our Phase 4 sample is unclear but may be related to facility closures during COVID restrictions. Reduced mobility limitations (7.2% vs. 6.4%), and social isolation (+ 0.8 vs. + 0.92) following program participation, were similar for Phase 3 compared with Phase 4, respectively.

**Correct Discussion #2**.

Between Phases 1–2 and 3 we observed a median ‘voltage drop’ or ‘scale-up penalty’ of 52.6% across outcomes [18]; however, we did not observe a further drop between Phases 3 and 4. This finding may be a function of slightly lower baseline PA in our Phase 4 sample (2.1 days/week; 95% CI: 2.0, 2.2) as compared with Phase 3 participants (2.5 days/week; 95% CI: 2.3, 2.6) allowing more room for change/improvements. The reason for lower baseline PA in our Phase 4 sample is unclear but may be related to facility closures during COVID restrictions. Reduced mobility limitations following program participation, were similar for Phase 3 (5.8%) compared with Phase 4 (6.0%), respectively.
