# Supplementary material for: Does optimizing Choose to Move – a health-promoting program for older adults – enhance scalability, program implementation and effectiveness?
Source: Int J Behav Nutr Phys Act. 2024 Dec 18;21:140. doi: 10.1186/s12966-024-01649-9 (PMC11657647; doi:10.1186/s12966-024-01649-9)
Supplement: Supplementary file 1 — Supplementary Material 1: Table S1; Table S2; Table S3. [file 12966_2024_1649_MOESM1_ESM.docx]

## **Supplementary Table 1.** Core functions (behaviour change techniques) from the behaviour change technique taxonomy v1^[[1]](#footnote-1)^) within Choose to Move Phase 3 and Phase 4.

|  | **Core functions (behaviour change techniques)** | |
| --- | --- | --- |
| **Intervention component** | **Phase 3** | **Phase 4** |
| One-on-one consultation | Activity coaches use the following behavior changes techniques with all CTM participants:   1. *Goals and planning*  - Goal setting (behaviour) (1.1) - Problem solving (1.2) - Action planning (1.4) - Commitment (1.9)   As the consultation is tailored to the participant’s needs, activity coaches may use some or all of the following behavior change techniques:   1. *Social support*  - Social support (unspecified) (3.1) - Social support (practical) (3.2) - Social support (emotional) (3.3)  1. *Shaping knowledge*  - Instruction on how to perform the behaviour (4.1)   *15. Self-belief*   - Verbal persuasion about capability (15.1) - Focus on past success (15.3) | Same as Phase 3 |
| Group meetings | 1. *Social support*  - Social support (unspecified) (3.1) - Social support (practical) (3.2) - Social support (emotional) (3.3)  1. *Shaping knowledge*  - Instruction on how to perform the behaviour (4.1)  1. *Natural consequences*  - Information about health consequences (5.1)  1. *Comparison of behaviour*  - Demonstration of the behaviour (6.1)   *8. Repetition and substitution*   - Behavioural practice/rehearsal (8.1) | Same as Phase 3 with the addition of:   1. *Goals and planning*  - Goal setting (behaviour) (1.1) - Problem solving (1.2) - Action planning (1.4) - Review behaviour goals (1.5) - Commitment (1.9)  1. *Feedback and monitoring*  - Self-monitoring of behavior (2.3)  1. *Repetition and substitution*  - Habit formation (8.3)   *15. Self-belief*   - Verbal persuasion about capability (15.1) |
| Telephone check-ins | 1. *Goals and planning*  - Goal setting (behaviour) (1.1) - Problem solving (1.2) - Action planning (1.4) - Review behaviour goals (1.5) - Commitment (1.9)  1. *Feedback and monitoring*  - Self-monitoring of behavior (2.3)  1. *Social support*  - Social support (unspecified) (3.1) - Social support (practical) (3.2) - Social support (emotional) (3.3)  1. *Shaping knowledge*  - Instruction on how to perform the behaviour (4.1)  1. *Repetition and substitution*  - Habit formation (8.3)   *15. Self-belief*   - Verbal persuasion about capability (15.1) | Not applicable – check-ins were removed for Phase 4.  Core functions associated with this component were moved to group meetings |

## **Supplementary Table 2.** TIDieR checklist for Choose to Move Phase 4.

| **Item No.** | **Item** |
| --- | --- |
| **Brief name** | Provide the name or a phrase that describes the intervention. |
| 1. | Choose to Move (CTM) – Phase 4 |
| **Why** |  |
| 2. | The health benefits of physical activity, and the health risks associated with loneliness, are well-documented. Despite this, most older adults do not meet recommended levels of physical activity and many are lonely. In addition, effective interventions must be scaled-up and delivered broadly in order to enhance health at the population level.  *Intervention:* CTM incorporates foundational elements of the Community Healthy Model Program for Seniors (CHAMPS) intervention which was successfully implemented and disseminated at an organizational level and improved health outcomes in participants [1, 2]. CHAMPS was based on principles derived from social cognitive theory [2]. CTM is a choice-based, coach and peer-supported model where participants choose physical activity regimens based on their personal activity preferences, health status, resources, and readiness to change. CTM incorporates elements common to CHAMPS and other successful interventions [3, 4]. We describe previous phases of CTM in detail, elsewhere [5-9].  *Implementation:* CTM is being scaled-up (since 2015) across British Columbia, Canada. A Central Support Unit (CSU) uses a suite of implementation strategies to support delivery partner organizations. We selected implementation strategies based on our guiding frameworks [10, 11] and our 10+ years experience scaling up health-promoting initiatives across BC (e.g., Action Schools! BC [12]). |
| **What** |  |
| 3. Materials | *Older adults:* Participants received the following materials to support their engagement in CTM: 1) Tell me about yourself worksheet; 2) Guided goal setting and action planning worksheet (intervention start); 3) Guided goal setting and action planning worksheets (intervention end – includes short- and long-term goals beyond CTM participation); 4) Physical activity log for tracking the type and amount of physical activity. One of our major delivery partners provided complimentary memberships to participants attending CTM through their facilities.  *Activity coaches:* coaches were provided with a slide deck and supporting resources for each group meeting. Each slide deck included an icebreaker activity, a discussion topic, movement breaks and guided discussion questions. Example discussion topics are goal setting, incidental physical activity, nutrition, falls prevention, and stress management.    *Recreation coordinators and recreation managers:* We provide an information package about the program to educate recreation centre staff (e.g., what is CTM, the role of recreation centre staff, participant eligibility requirements), resources to support participant recruitment and registration packages to provide to interested participants. |
| 4. Procedures | *Implementation*   - Program implementation is supported by the CSU. The CSU works with delivery partner organizations to create delivery agreements, train activity coaches, provide resources (e.g. activity coach job descriptions, promotion and recruitment materials), and provides ongoing support and consultation as needed. - Activity coaches receive comprehensive training (~8hr) on all aspects of CTM (e.g., program logistics, content, evaluation) prior to delivery. Training was delivered online for Phase 4. Refresher training modules were offered to coaches who previously completed training for Phases 1-3.   *Intervention*   - Older adults register for the CTM program through their local recreation centre/delivery partner organization. - Activity coaches (hired by delivery partner organizations) offer the following two program components to registered participants: a 30-minute one-on-one consultation and 8 group meetings with other older adult participants. |
| **Who provided** |  |
| 5 | All components of CTM are delivered by activity coaches hired by our community-based delivery partners. At large partner organizations, activity coaches are trained fitness leaders with older adult specialization or kinesiologists. All activity coaches complete CTM training (~8 hr in-person or online with refresher/update training as needed) prior to delivering CTM.  Recreation managers and coordinators are frontline staff at community centres who interface with prospective participants about the program, facilitate participant registration and arrange room bookings as required. |
| **How** |  |
| 6 | Delivery modes were:   - One-on-one consultation: face-to-face or virtual (internet or phone) as COVID restrictions permitted (individual) - Group meetings: face-to-face or virtual (internet or phone) as COVID restrictions permit (group) |
| **Where** |  |
| 7 | CTM is delivered across British Columbia, Canada. In Phase 4 CTM is delivered in-person or virtually (Zoom, Go to Meeting) as COVID restrictions and community preferences dictated. |
| **When and How Much** | |
| 8 | - One-on-one consultation: 30-min - Group meetings: 8 motivational group meetings (60-min each) over 3 months |
| **Tailoring** |  |
| 9 | The CSU works with delivery partner organizations to tailor implementation strategies as needed to support delivery. The CSU meets with partner organizations as needed to support program delivery.  CTM participants work with their activity coach to set goals and tailor a personalized action plan that aligns with their interests, abilities and available resources. |
| **Modifications** |  |
| 10 | The CSU works with delivery partner organizations to support delivery. Implementation strategies are tailored as needed for each organization  Modifications made by activity coaches for their groups included: adding or removing movement breaks, removing ice-breaker activities, modifying group meeting formats (e.g. using breakout rooms, meeting outdoors), sourcing and sharing additional relevant resources, sending additional email reminders etc. |
| **How well** |  |
| 11 Planned | We assessed dose-delivered (number of group meetings delivered), fidelity to core functions (categorical: at no (0), a few (1-2), some (3-5), most (6-7) or all (8) group meetings and adaptation (text response) via activity coach survey at the end of the program. |
| 12 Actual | Activity coaches delivered all (8/8) group meetings in 134/135 programs. Fidelity to core functions was 96-100%. Adaptations included adding or removing movement breaks, removing icebreakers, less focus on peer check-ins, changes to group meeting format (e.g., using breakout rooms in virtual meetings, meeting outdoors), sharing additional resources relevant to the group, additional contact (e.g. email reminders) in between group meetings. |

*Note.* CHAMPS = Community Healthy Activities Model Program for Seniors; CTM = Choose to Move

## **Supplementary Table 3.** Baseline socio-demographic characteristics of participants who completed the Choose to Move program and those lost to follow-up. Values are n (%) or mean (standard deviation). Sample size varies due to missing data.

|  | **Completed CTM & evaluation (3 months)** | **Lost to follow-up (at 3 months)** | **p-value** |
| --- | --- | --- | --- |
| Participants, n (men / women / prefer not to answer) | 940 (110/ 826 / 4) | 186 (18 / 168 / 0) | 0.416 |
| % (men) | 11.8% | 9.7% |  |
| Age, mean (SD) | 71.6 (6.0) | 71.2 (6.0) | 0.406 |
| Age category, n (%) |  |  |  |
| <75 years | 649 (70.5) | 135 (74.2) | 0.313 |
| >75 | 272 (29.5) | 47 (25.8) |  |
| Delivery partner, n (BCRPA / YMCA) | 646 / 294 | 128 / 58 | 0.980 |
| BMI, kg/m^2^, mean (SD) | 30.3 (6.9) | 29.8 (6.6) | 0.482 |
| Ethnicity, n (%) |  |  |  |
| White | 834 (90.2) | 158 (86.8) | 0.366 |
| Asian | 57 (6.2) | 14 (7.7) |  |
| Other | 34 (3.7) | 10 (5.5) |  |
| Educational attainment, n (%) |  |  |  |
| Secondary or less | 157 (17.0) | 39 (21.4) | 0.223 |
| Some trade, technical school or college | 291 (31.5) | 48 (26.4) |  |
| Some university | 476 (51.5) | 95 (52.2) |  |
| Chronic Conditions, n (%) |  |  |  |
| 0 | 408 (43.9) | 84 (45.4) | 0.922 |
| 1 | 269 (29.0) | 53 (28.7) |  |
| ≥ 2 | 252 (27.1) | 48 (26.0) |  |
| Mobility limitations (walk and/or stair), n (%) |  |  |  |
| Yes | 373 (40.0) | 82 (44.1) | 0.303 |
| No | 559 (60.0) | 104 (55.9) |  |
| Self-rated health, n (%) |  |  |  |
| Very poor, poor or fair for age | 539 (58.1) | 104 (55.9) | 0.585 |
| Good or excellent for age | 389 (41.9) | 82 (44.1) |  |
| Physical activity (# days/week > 30 min), mean (SD) | 2.1 (2.0) | 2.1 (1.9) | 0.900 |
| Mobility (MAT-sf score), mean (SD) | 51.3 (10.3) | 51.4 (10.6) | 0.980 |
| Social isolation (score, 0–20), mean (SD) | 12.1 (3.9) | 11.6 (4.0) | 0.137 |
| Loneliness (score, 3–9), mean (SD) | 5.2 (1.8) | 5.4 (2.0) | 0.188 |

British Columbia Parks and Recreation Association; YMCA: Young Men’s Christian Association

1. Stewart, A.L., et al., *Physical activity outcomes of CHAMPS II: a physical activity promotion program for older adults.* Journals of Gerontology - Series A Biological Sciences and Medical Sciences, 2001. **56**(8): p. M465-70.

2. Stewart, A.L., et al., *Evaluation of CHAMPS, a physical activity promotion program for older adults.* Annals of Behavioral Medicine, 1997. **19**(4): p. 353-61.

3. Chase, J.D., *Interventions to increase physical activity among older adults: A meta-analysis.* Gerontologist, 2015. **55**(4): p. 706-18.

4. Gilchrist, H., et al., *Use of behavior change techniques in physical activity programs and services for older adults: findings from a rapid review.* Ann Behav Med, 2024. **58**(3): p. 216-226.

5. McKay, H.A., et al., *Translational formative evaluation before scale-up of a physical activity intervention for older men.* Translational Journal of the American College of Sports Medicine, 2019. **4**(14): p. 106-113.

6. Mackey, D.C., et al., *Men on the Move: A randomized controlled feasibility trial of a scalable, choice-based physical activity and active transportation intervention for older men.* Journal of Aging and Physical Activity, 2019. **27**: p. 489-502.

7. McKay, H.A., et al., *Implementation of a co-designed physical activity program for older adults: positive impact when delivered at scale.* BMC Public Health, 2018. **18**(1): p. 1289.

8. Gray, S.M., et al., *Getting Ready for Scale-Up of an Effective Older Adult Physical Activity Program: Characterizing the Adaptation Process.* Prevention Science, 2020.

9. McKay, H.A., et al., *What is the 'voltage drop' when an effective health promoting intervention for older adults-Choose to Move (Phase 3)-Is implemented at broad scale?* PLoS One, 2023. **18**(5): p. e0268164.

10. Durlak, J.A. and E.P. DuPre, *Implementation matters: a review of research on the influence of implementation on program outcomes and the factors affecting implementation.* American Journal of Community Psychology, 2008. **41**(3-4): p. 327-50.

11. Yamey, G., *Scaling up global health interventions: a proposed framework for success.* PLoS Medicine, 2011. **8**(6): p. e1001049.

12. Nettlefold, L., et al., *Scaling up Action Schools! BC: How Does Voltage Drop at Scale Affect Student Level Outcomes? A Cluster Randomized Controlled Trial.* International Journal of Environmental Research and Public Health, 2021. **18**(10): p. 5182.

1. Michie S, Richardson M, Johnston M, Abraham C, Francis J, Hardeman W, et al. The behavior change technique taxonomy (v1) of 93 hierarchically clustered techniques: building an international consensus for the reporting of behavior change interventions. Ann Behav Med. 2013;46(1):81-95. [↑](#footnote-ref-1)
